# Efficacy of Phototherapy With 308-nm Excimer Light for Skin Microbiome Dysbiosis and Skin Barrier Dysfunction in Canine Atopic Dermatitis

**DOI:** 10.3389/fvets.2021.762961

**Published:** 2021-12-03

**Authors:** Ju-Yong Park, Seon-Myeong Kim, Jung-Hyun Kim

**Affiliations:** ^1^Department of Veterinary Internal Medicine, College of Veterinary Medicine, Konkuk University, Seoul, South Korea; ^2^KR Lab Bio Incorporation, Seoul, South Korea

**Keywords:** canine atopic dermatitis (CAD), skin microbiome, skin barrier function, phototherapy (PTT), excimer light

## Abstract

The management of canine atopic dermatitis, an allergic skin disorder, is challenging. To investigate the effect of phototherapy using a 308-nm excimer light as a topical treatment for canine atopic dermatitis, 10 dogs with canine atopic dermatitis and 10 with non-allergic skin were enrolled in this study. Phototherapy was applied every 7 days for a total of 2 months. The skin microbiome, skin barrier function, and clinical outcomes were evaluated after phototherapy. Phototherapy significantly changed the composition of the skin microbiome of dogs with atopic dermatitis and significantly increased the relative abundance of the phyla Actinobacteria and Cyanobacteria. It significantly alleviated the clinical signs of canine atopic dermatitis without serious adverse effects. Transepidermal water loss, as a measure of skin barrier function, significantly decreased after phototherapy. In addition, phototherapy increased microbial diversity and decreased the relative abundance of *Staphylococcus pseudintermedius* associated with the severity of canine atopic dermatitis. These results suggest that the excimer light therapy is a suitable and safe therapeutic option for canine atopic dermatitis, which is also a spontaneous animal model of atopic dermatitis.

## Introduction

Canine atopic dermatitis (cAD) is a chronic allergic dermatitis that affects approximately 10–15% of dogs (1, 2). The International Committee for Allergic Diseases of Animals has recommended guidelines for the management of cAD. The most effective treatment options include cyclosporin, glucocorticoid, oclacitinib, bathing, and allergen-specific immunotherapy (3). Additionally, lokivetmab (an injectable anti-canine interleukin-31 [IL-31] monoclonal antibody) has been used as a new treatment option for atopic dermatitis (AD) (1). However, there is no specific treatment protocol for cAD, and the choice of treatment depends on the response to treatment, potential side effects, owner compliance, and medication costs (2).

Phototherapy is one of several treatments that effectively alleviates inflammatory lesions of the skin without systemic side effects in refractory human AD (4). Excimer light, which is a narrow-band UVB light at a 308-nm wavelength, acts topically. It can be safely applied as it has few known side effects, and its effectiveness has been proven for many human skin diseases such as plaque psoriasis and vitiligo (4–6). In recent studies, excimer light treatment was used effectively in human AD patients (4, 7).

In dogs, a normal skin microbiome is important for maintaining normal skin function as it coordinates the innate immune response and prevents bacterial infection and proliferation (8). Dysbiosis of the skin microbiome was observed in cAD lesions (9). In addition to changes in the composition of the skin microbiome, cAD is caused by skin barrier dysfunction, and the disease worsens the skin barrier with chronicity. Antimicrobial treatment can induce normalization of the skin microbiome and barrier function in cAD (10).

This study aimed to investigate the clinical effects of phototherapy using an excimer light in cAD and to examine any associated changes in the skin microbiome and skin barrier function.

## Materials and Methods

### Subjects

This study was approved by the KonKuk University Institutional Animal Care and Use Committee (approval number: KU20057). Each owner provided informed consent for their dog to participate in the study.

#### Allergic Group

Client-owned dogs with cAD (*n* = 10) participated in this study from April 2020 to March 2021 at the Konkuk University Veterinary Medical Teaching Hospital, Korea. Dogs with cAD fulfilled standard criteria which included at least five criteria of Favrot's criteria (11). Ectoparasitic diseases were ruled out based on multiple skin scrapings, and all dogs received monthly flea prevention treatments. All dogs performed an elimination diet trial for 8 weeks using a commercialized novel or hydrolyzed protein diet to exclude food allergy. Dogs with a partial response to the elimination diet trial were included in this study. Sensitization to environmental allergens was confirmed in all dogs based on the results of a serum-specific IgE antibody test (A-vetech Co., Seoul, South Korea). The use of cyclosporine or systemic glucocorticoid was permitted if used for at least 4 months, and the use of oclacitinib or lokivetmab was permitted if used for at least 3 months, with the dose remaining unchanged. Medicated shampoo was permitted if used for at least 1 month, with the number of applications remaining unchanged (once a week) (12). Dogs receiving any antimicrobial/antifungal therapy, glucocorticoids, cyclosporine, or antihistamine and anti-inflammatory therapy within 4 weeks were excluded.

#### Control Group

Healthy dogs (*n* = 10) were included in this study if they had no history or clinical findings of allergic skin disorders and had not received anti-inflammatory, antibiotic, or immunosuppressive drugs for at least 4 months before participation.

### Protocol With Excimer Light Therapy

**Figure 1:**
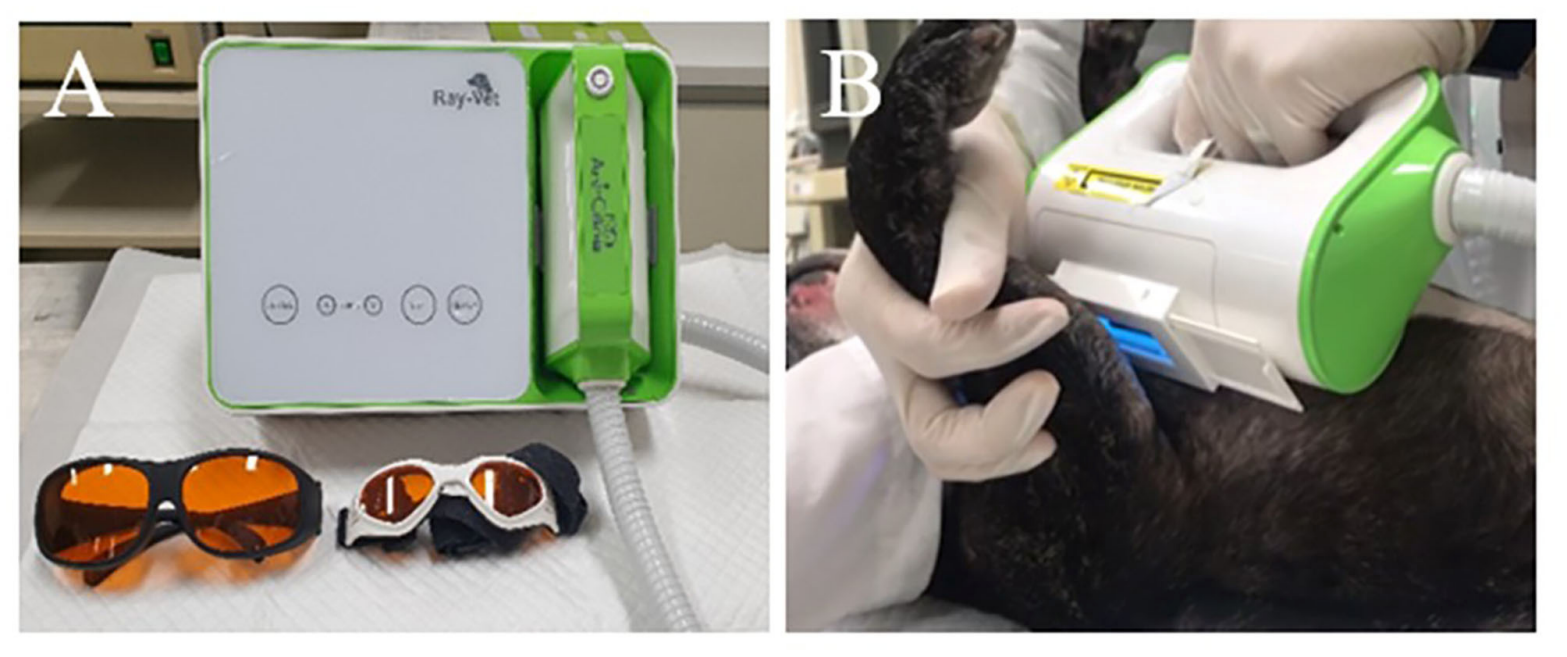
UVB-light skin therapy system for animals and application of phototherapy with 308-nm excimer light. **(A)** 308-nm excimer light delivery machine with UV-blocking goggles for humans and animals (Ray-Vet, Lameditech Corporation, Seoul, Korea). **(B)** A patient with canine atopic dermatitis receives phototherapy with 308-nm excimer light at the axillary region.

Excimer light therapy was performed with a UVB-light skin therapy system for animals (Ray-Vet, Lameditech Corporation, Seoul, Korea, Figure 1). The 308-nm excimer light was delivered via a handpiece. The beam diameter was 2 × 4.5 cm^2^. The excimer light therapy was applied to all atopic skin lesions, including those in the ear pinna, axilla, flanks, front paws, hind paws, abdomen, and groin. However, mucocutaneous areas (lips, perineum) were excluded in the allergic group. The excimer light was applied at each site for 30 s, with the excimer light being applied twice or more to the abdomen. In large breeds, the excimer light was applied twice or more to each site. The initial irritation dose was 300 mJ/cm^2^. The dose was increased by 150 mJ/cm^2^ every 2 weeks, and the largest dose was 750 mJ/cm^2^. The therapy was repeated every 7 days for 2 months. The side effects (erythema, blistering, hyperpigmentation, skin cancer, pruritus, burning) of phototherapy observed in human AD patients were evaluated every week until 1 month after the end of the excimer light therapy, and if side effects occurred during the excimer light therapy session, the dose was not increased (13). The dogs wore UV-blocking goggles to protect their eyes. A detailed protocol is described in Table 1.

**Table 1:** Excimer light therapy protocol.

**Week**	**Dose**
Week 1, 2	Total 300 mJ/cm^2^ (10 mW/cm^2^ for 30 s)
Week 3, 4	Total 450 mJ/cm^2^ (15 mW/cm^2^ for 30 s)
Week 5, 6	Total 600 mJ/cm^2^ (20 mW/cm^2^ for 30 s)
Week 7, 8	Total 750 mJ/cm^2^ (25 mW/cm^2^ for 30 s)

### Assessment of Clinical Efficacy

The severity of cAD was evaluated using the CADESI-4, which is a validated method designed to assess the severity of cAD quickly and accurately. In the CADESI-4, cAD skin lesions were scored from 0 to 3 for body sites where AD mainly occurs (14). CADESI-4 for the allergic group was assessed by the same veterinarian unless relevant to this study. The degree of pruritus was evaluated using the Pruritus Visual Analog Scale (PVAS), a verified scale for assessment by the owners. The owner rates pruritus on a scale of 0 to 10 using the PVAS (15). The clinical efficacy of excimer light therapy was evaluated before the start of therapy and at the end of therapy using both the CADESI-4 and PVAS in the allergic group.

### Assessment of Skin Barrier Function

Transepidermal water loss (TEWL) and stratum corneum hydration (SCH) were evaluated using a GPskin Barrier® (Gpower corporation, Seoul, Korea). TEWL and SCH were assessed before the start of excimer light therapy, after four sessions of excimer light therapy, and at the end of excimer light therapy in the ear pinna, axilla, and groin after hair clipping in the allergic group. In the control group, TEWL and SCH were measured once in the same skin area. All dogs were measured in the same area where the room temperature was 21–25°C, and the relative humidity was 40–50%. Five successive measurements were performed, and the mean values were calculated.

### Microbiome Sample Collection

Samples were collected from three sites (right ear pinna, right axilla, right groin) after hair clipping. Samples from the control group were collected once. Samples from the allergic group were collected three times before the start of excimer light therapy, after four sessions of excimer light therapy, and at the end of excimer light therapy. All dogs were bathed at least 7 days before sample collection. Samples were obtained using a sterile culture swab applicator (eSwab™, COPAN Diagnostics, Murrietta, CA). Each swab applicator was soaked in sterilized normal saline and swabbed on the skin 40 times, while each swab was rotated by one-quarter every 10 times (8). The swabs were stored at −80°C until further analysis.

### DNA Extraction and *16S* rRNA Sequencing

Bacterial DNA extraction was performed using a DNeasy PowerSoil kit (Qiagen, Hilden, Germany) according to the manufacturer's instructions. To analyze the skin microbiome, the hypervariable V1-3 region of the 16S ribosomal RNA (rRNA) gene was amplified. The primer sets for gene amplification are described in Supplementary Table 1. Polymerase chain reactions (PCR) was performed using the KAPA HiFi HotStart ReadyMix (Roche KAPA Biosystems, Cape Town, South Africa) according to the manufacturer's recommendations. The PCR products were used for the construction of 16S rRNA gene libraries, following the MiSeq System guidelines (Illumina Inc., San Diego, CA). Subsequently, sequencing (600 cycles, paired end) was performed using the MiSeq System (Illumina, Inc., San Diego, CA) according to the manufacturer's recommendations.

### Bacterial *16S* rRNA Gene Analysis

The demultiplexing step of the Illumina MiSeq paired-end sequencing (2 × 300 bp) generates two FastQ files (one forward and other reverse) for each sample. These FastQ files were assessed using FASTQC (16) and were subsequently analyzed via the DADA2 pipeline (ver. 1.18.0) (17) within the R statistical computing environment (ver. 4.0.3; https://www.r-project.org/) for filtering, dereplication, calculating error rates, merging sequences, making an amplicon sequence variants (ASV) table, and removing chimeras. First, this study used the filterAndTrim function with the following parameters: truncLen = c (300, 250) and trimLeft = 20 and default setting for others. The primer length for forward and reverse reads was approximately 20 bp. Thus, 20 bp was removed from the start of each read. The errors rates were calculated and removed from the dereplicated reads. While this study used the MergePairs function with default justConcatenate = FALSE option, some merged reads were shorter than expected for the V1–V3 region. This study collected these short, merged reads and merge-failed reads that had few overlapping parts, and re-attempted merging with the justConcatenate = TRUE option which concatenates reads by inserting Ns between them. Thus, the length of all merged reads exceeded 470 bp. A sequence table was constructed from the merged reads, and chimeras were removed using the removeBimeraDenovo function. Finally, taxonomy was assigned to cleaned ASVs using the naïve RDP Bayesian classifier (ver. 16) with the Phyloseq (ver. 1.34.0) package (18). Demultiplexed FastQ files were deposited in the Sequence Read Archive of the National Center for Biotechnology Information (https://www.ncbi.nlm.nih.gov/sra; accession number PRJNA739506).

### Statistical Analysis

Statistical analysis was performed using SPSS software (IBM Corporation, Armonk, NY). Student's *t*-tests or non-parametric Mann–Whitney tests were used to compare the differences between the control and allergic groups. To compare the effects of excimer light therapy, paired *t*-tests or non-parametric Wilcoxon's rank-sum tests were used in the allergic group. Pearson's correlation tests were conducted to analyze the correlations between the changes in the skin microbiome and those in the CADESI-4, PVAS score, TEWL, and SCH. Data are expressed as the mean ± standard deviation.

## Results

### Characteristics of Study Subjects

The mean ages of the dogs in the allergic and control groups were 7.7 ± 3.1 years and 6.8 ± 4.8 years, respectively. In the allergic group, six dogs were treated with excimer light monotherapy, three with excimer light therapy combined with oclacitinib, one with excimer light therapy combined with lokivetmab, with none of the dogs being treated with cyclosporine or systemic glucocorticoid. None of the dogs were administered any type of antibiotics or glucocorticoids for 4 weeks prior to the excimer light therapy. Medicated shampoo was used in combination in two dogs and had been used for at least 4 months prior to the excimer light therapy. The total excimer light therapy time was 7–15 min depending on the body size. Mild erythema of the size of the beam diameter without pruritus occurred in two dogs after the first session of excimer light therapy. This mild adverse effect resolved spontaneously, and when two dogs came to the hospital for the second session of excimer light therapy, the lesion could not be observed. Detailed information on the study subjects is provided in Tables 2, 3.

**Table 2:** Signalment information.

**ID**	**Group**	**Breed**	**Age**	**Sex**	**Food allergy**	**Environmental allergens**	**Seasonality of pruritus**
1	Control	CKCS	2 Y	CM	N	NA	NA
2	Control	Fox terrier	8 Y	CM	N	NA	NA
3	Control	Yorkshire terrier	10 Y	SF	N	NA	NA
4	Control	Yorkshire terrier	16 Y	SF	N	NA	NA
5	Control	Yorkshire terrier	7 Y	CM	N	NA	NA
6	Control	Daschund	2 Y	CM	N	NA	NA
7	Control	Daschund	1 Y	CM	N	NA	NA
8	Control	Maltese	11 Y	CM	N	NA	NA
9	Control	Mixed	3 Y	CM	N	NA	NA
10	Control	Maltese	8 Y	CM	N	NA	NA
11	Allergy	Fox terrier	8 Y	SF	N	Mite(*Tyrophagus putrescentiae*)	NS
12	Allergy	Maltese	12 Y	CM	N	Mite(*Acarus siro*)	NS
13	Allergy	Poodle	8 Y	SF	N	Mite(*Dermatophagoides pteronyssinus*)	NS
14	Allergy	Poodle	3 Y	CM	Partial	Fungi(*Alternaria*)	S
15	Allergy	Maltese	11 Y	CM	Partial	Fungi(*Alternaria*)	NS
16	Allergy	French bulldog	8 Y	M	N	Mite(*Dermatophagoides pteronyssinus*)	NS
17	Allergy	Golden retriever	6 Y	CM	Partial	Fungi(*Alternaria*)	NS
18	Allergy	Mixed	7 Y	CM	Partial	Mite(*Tyrophagus putrescentiae*)	S
19	Allergy	Pekinese	11 Y	F	Partial	Fungi(Alternaria)	S
20	Allergy	Standard poodle	3 Y	CM	N	Mite(*Tyrophagus putrescentiae*)	NS

*CKCS, cavalier king charles spaniel; CM, castrated male; F, female; N, none; NA, not available; NS, non-seasonality; M, male; S, seasonality; SF, spayed female; Y, years*.

**Table 3:** Medical history of the allergic group.

**ID**	**Allergy treatment**		**Medicated shampoo**		**Antibiotics**		**Glucocorticoid**	
	**Types**	**Duration of use**	**Types**	**Duration of use**	**Types**	**Duration of discontinue**	**Types**	**Duration of discontinue**
11	N	NA	N	NA	N	NA	N	NA
12	Oclacitinib	>2 years	N	NA	N	NA	N	NA
13	N	NA	N	NA	Systemic (cephalexin)	2 months	N	NA
14	Lokivetmab	>4 months	2% chlorhexidine 2% miconazole	> 4 months	N	NA	N	NA
15	N	NA	N	NA	N	NA	N	NA
16	Oclacitinib	>1 years	N	NA	N	NA	Topical (hydrocortisone ointment)	3 months
17	Oclacitinib	>1 years	N	NA	N	NA	Systemic (PDS 0.5 mg/kg PO SID)	5 weeks
18	N	NA	N	NA	Systemic (lincomycin)	2 months	Systemic (PDS 0.5 mg/kg PO SID)	4 months
19	N	NA	2% chlorhexidine 2 % miconazole	> 1 year	N	NA	Systemic (PDS 0.5 mg/kg PO SID)	> 2 years
20	N	NA	N	NA	N	NA	N	NA

*N, none; NA, not available; PDS, prednisolone*.

### Clinical Efficacy of Excimer Light Therapy

**Figure 2:**
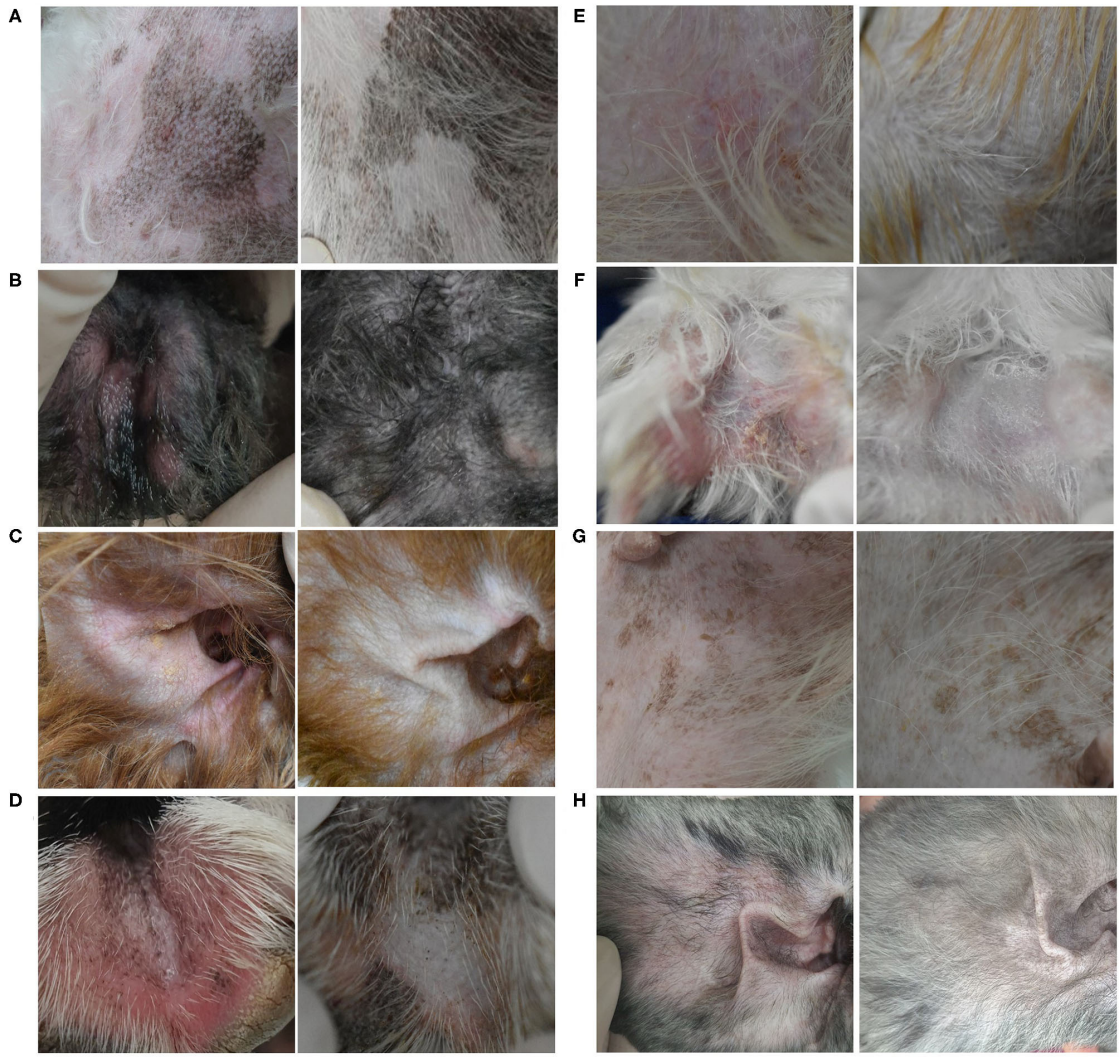
Clinical efficacy of excimer light therapy. Clinical signs of canine atopic dermatitis were improved after 8 weeks of excimer light therapy in the allergic group. **(A)** Dog 11, groin. **(B)** Dog 13, hind paws. **(C)** Dog 14, ear pinnae. **(D)** Dog 16, front paws. **(E)** Dog 17, groin. **(F)** Dog 18, hind paws. **(G)** Dog 19, groin. **(H)** Dog 20, ear pinnae [**(Left)**, before excimer light therapy; **(Right)**, after excimer light therapy].

**Figure 3:**
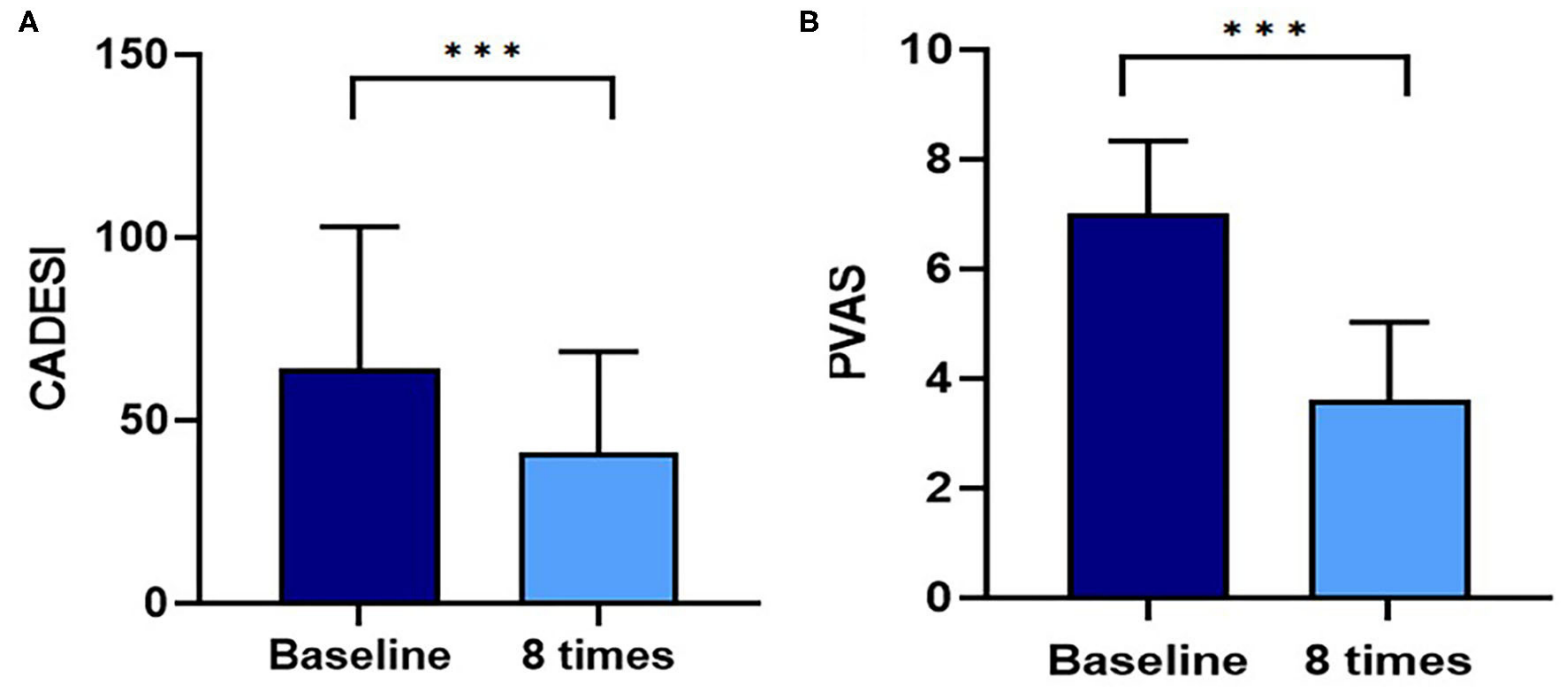
Assessment of the CADESI and PVAS with excimer light therapy. **(A)** Change in the CADESI with excimer light therapy. **(B)** Change in the PVAS with excimer light therapy. Eight times, end of the excimer light therapy in allergic group; baseline, before start of excimer light therapy in allergic group. CADESI, canine atopic dermatitis extent and severity index; PVAS, Pruritus Visual Analog Scale. All values are expressed as mean ± standard error. ^***^P < 0.001.

A flare, i.e., clinical signs of cAD, such as erythema, improved after 8 weeks of excimer light therapy in the allergic group (Figure 2). CADESI-4 decreased significantly after excimer light therapy in the allergic group, from 64.3 ± 38.6 to 41.0 ± 27.8 (*P* < 0.001; Figure 3A). PVAS score also significantly decreased after excimer light therapy in the allergic group, from 7.0 ± 1.3 to 3.6 ± 1.4 (*P* < 0.001; Figure 3B). In analyzing the mean improvement of the CADESI-4 after excimer light therapy, the mean decrease was slightly larger in the case of combination with other allergy treatment than in the case of excimer light monotherapy alone. When classified using CADESI-4 severity, the mean decrease was greatest in the order of moderate, mild, and severe cAD. In analyzing the mean improvement of PVAS after excimer light therapy, the mean decrease was greater in the case of combination with other allergy treatments than in the case of excimer light monotherapy alone. When classified using CADESI-4 severity, the mean decrease was greatest in the order of mild, moderate, and severe cAD (Table 4). The CADESI-4 and PVAS scores of each subject are presented in Supplementary Table 2.

**Table 4:** Clinical efficacy of excimer light therapy in the allergic group.

	**Combination therapy with allergy treatment**			**Monotherapy of excimer light therapy**			**CADESI severity**								
	**Pre**	**Post**	**%**	**Pre**	**Post**	**%**	**Mild**			**Moderate**			**Severe**		
							**Pre**	**Post**	**%**	**Pre**	**Post**	**%**	**Pre**	**Post**	**%**
n		4			6		3			3			4		
Mean CADESI	51	32	37.3	73	47	35.6	27.7	16.3	41.2	44.3	24	45.8	106.5	72.3	32.1
Mean PVAS	7	3.3	52.9	7	3.8	45.7	7.3	3	58.9	8.3	4.7	43.4	5.8	5.8	43.1

*CADESI, canine atopic dermatitis extent and severity index; N, number; Post, after eight session of the excimer light therapy in allergic group; Pre, before start of the excimer light therapy in allergic group; PVAS, Pruritus Visual Analog Scale*.

### Assessment of Skin Barrier Function

**Figure 4:**
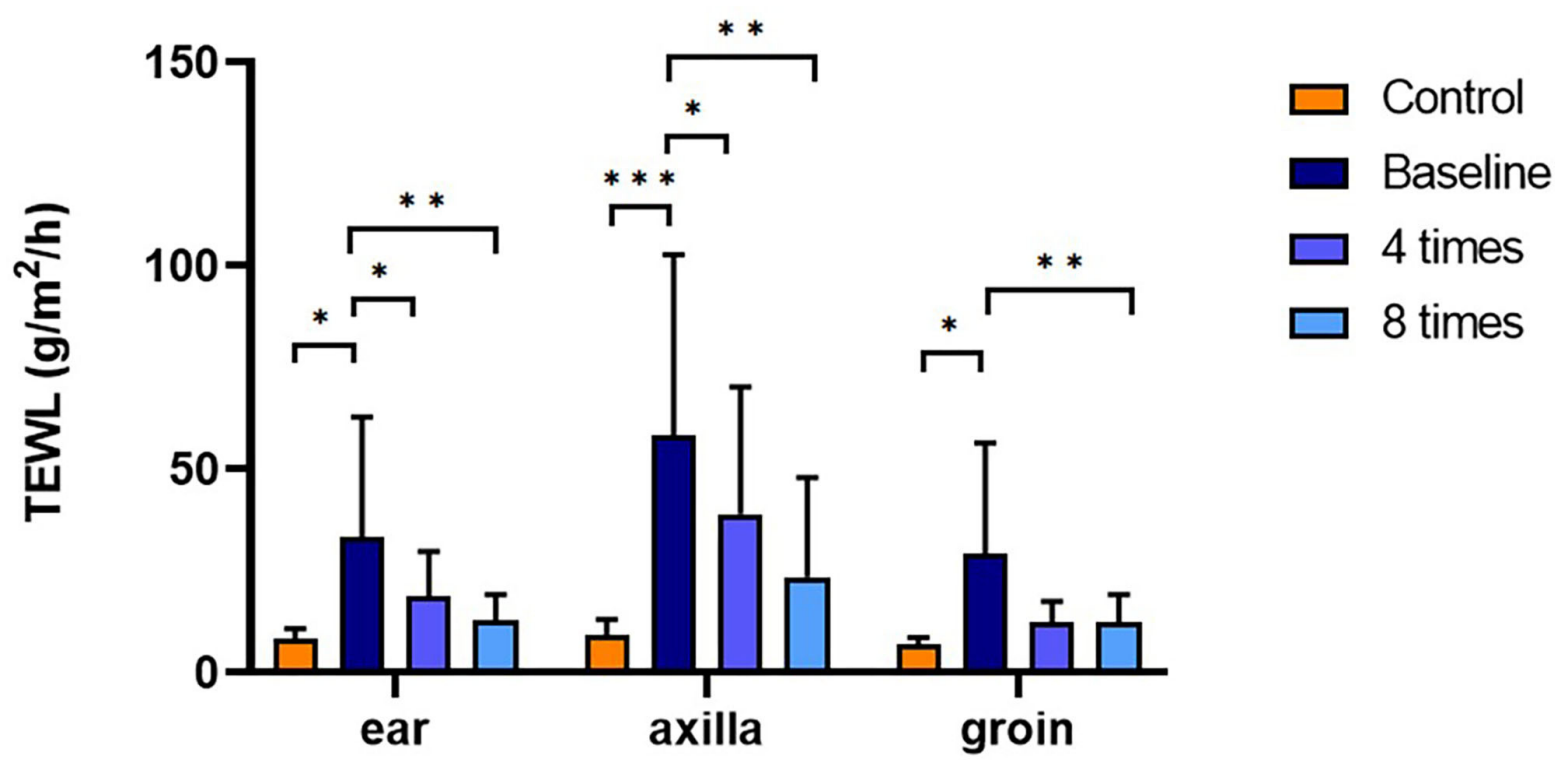
Assessment of TEWL with excimer light therapy. Difference in the TEWL between the control and allergic groups and change in the TEWL in the allergic group with excimer light therapy. Four times, fourth session of excimer light therapy in allergic group; eight times, end of the excimer light therapy in the allergic group; baseline, before the start of excimer light therapy in the allergic group; TEWL, transepidermal water loss. All values are expressed as mean ± standard error. ^*^P < 0.05, ^**^P < 0.01, ^***^P < 0.001.

**Figure 5:**
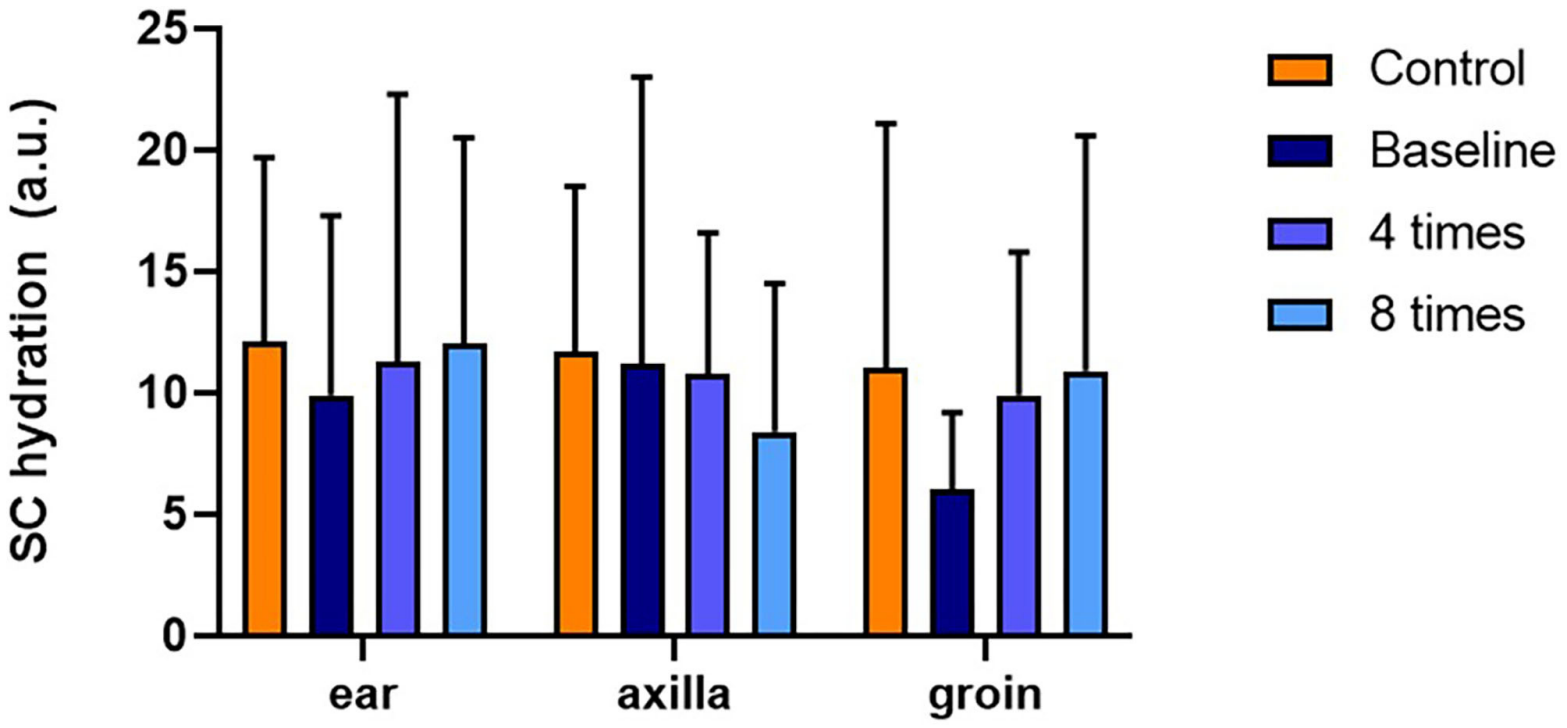
Assessment of SCH with excimer light therapy. Difference in the SCH between the control and allergic groups and change in the SCH in the allergic group with excimer light therapy. Four times, fourth session of excimer light therapy in allergic group; eight times, end of the excimer light therapy in the allergic group; a.u., arbitrary unit; baseline, before the start of excimer light therapy in the allergic group; SCH, stratum corneum hydration. All values are expressed as means ± standard error.

TEWL was significantly higher in the allergic group compared with that in the control group at all measurement sites (ear pinnae, *P* < 0.01; axilla, *P* < 0.001; groin, *P* < 0.001; Figure 4). Excimer light therapy significantly decreased the TEWL in the allergic group at all measurement sites (ear pinnae, *P* < 0.01; axilla, *P* < 0.01; groin, *P* < 0.01; Figure 4). In the ear pinnae and axilla, the TEWL significantly decreased after four sessions of excimer light therapy (ear pinnae, *P* < 0.05; groin, *P* < 0.05; Figure 4). No statistically significant difference was seen in SCH between the control and allergic groups. Likewise, SCH did not change significantly after excimer light therapy in the allergic group (Figure 5). The skin barrier function of each subject is stated in Supplementary Table 2.

### Analysis of the Skin Microbiome at the Phylum Level

**Figure 6:**
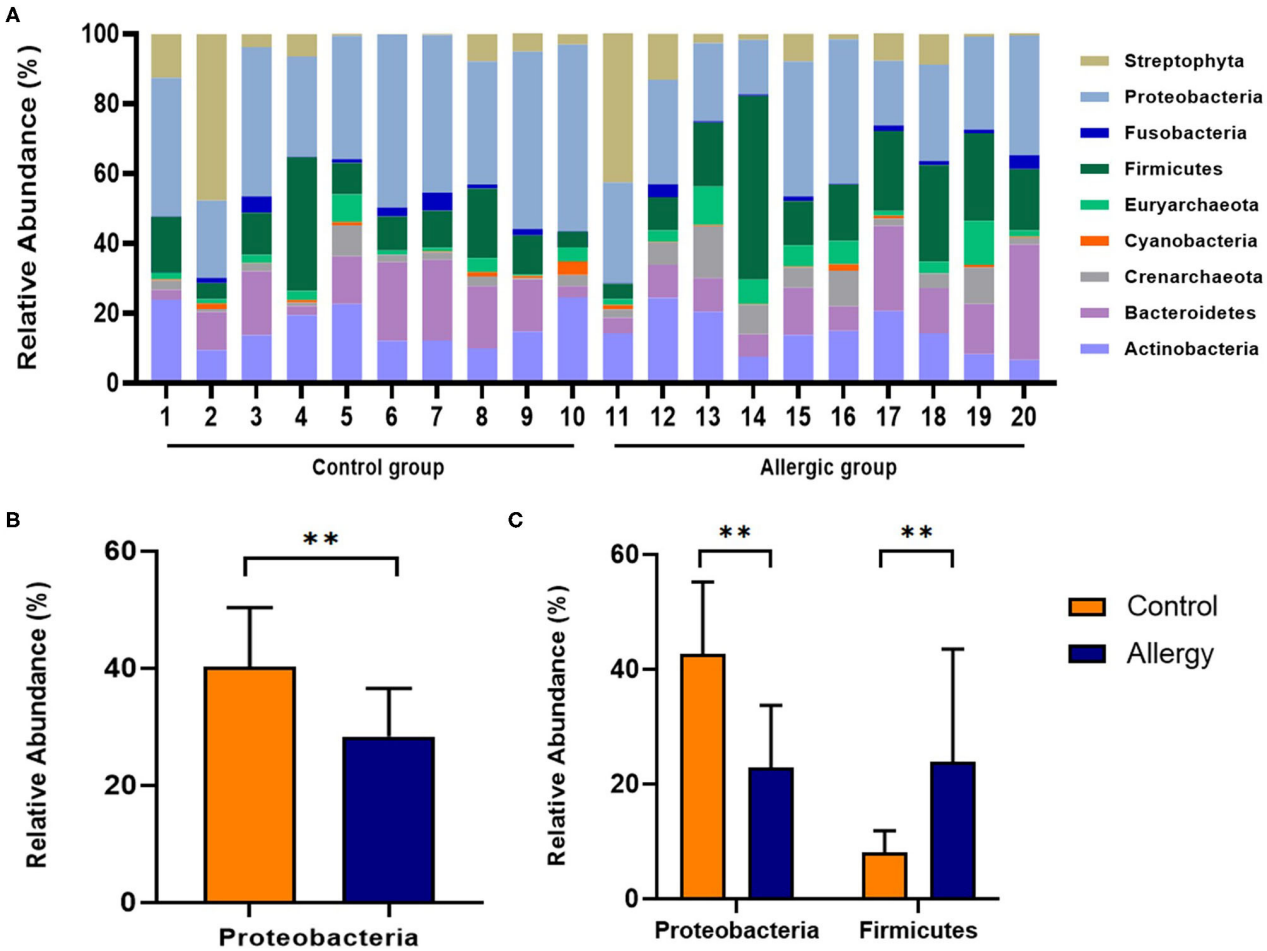
Analysis of the skin microbiome at the phylum level. **(A)** Individual composition of bacterial phyla in the control and allergic groups. **(B)** Statistical analysis of the mean relative abundance of the phylum Proteobacteria in axillary region. **(C)** Statistical analysis of the relative abundance of the phyla Firmicutes and Proteobacteria in the axillary region. All values are expressed as mean ± standard error. ^**^P < 0.01.

At all measurement sites, the most abundant phylum detected in the control group was Proteobacteria, followed by Actinobacteria, Firmicutes, and Bacteroidetes (40.3, 16.3, 13.6, 13.0%, respectively). Similarly, in the allergic group, the most abundant phylum detected was Proteobacteria, followed by Firmicutes, Actinobacteria, and Bacteroidetes (28.4, 20.7, 14.5, 13.6%, respectively). The second most abundant phylum was different between groups. Compared to the mean relative abundance of the phylum in all analyzed sites, the phylum Proteobacteria was significantly less abundant in the allergic group compared with that in the control group (allergic group, 28.4 ± 8.2%, control group, 40.3 ± 10.1%, *P* < 0.01; Figure 6). Specifically, in the axillary region, the phylum Firmicutes was more abundant, and the phylum Proteobacteria was less abundant in the allergic group than in the control group (Firmicutes, allergic group, 23.8 ± 19.7%, control group, 8.1 ± 3.8%; *P* < 0.01; Proteobacteria, allergic group, 22.8 ± 11.0%, control group, 42.7 ± 12.5%, *P* < 0.01; Figure 6).

**Figure 7:**
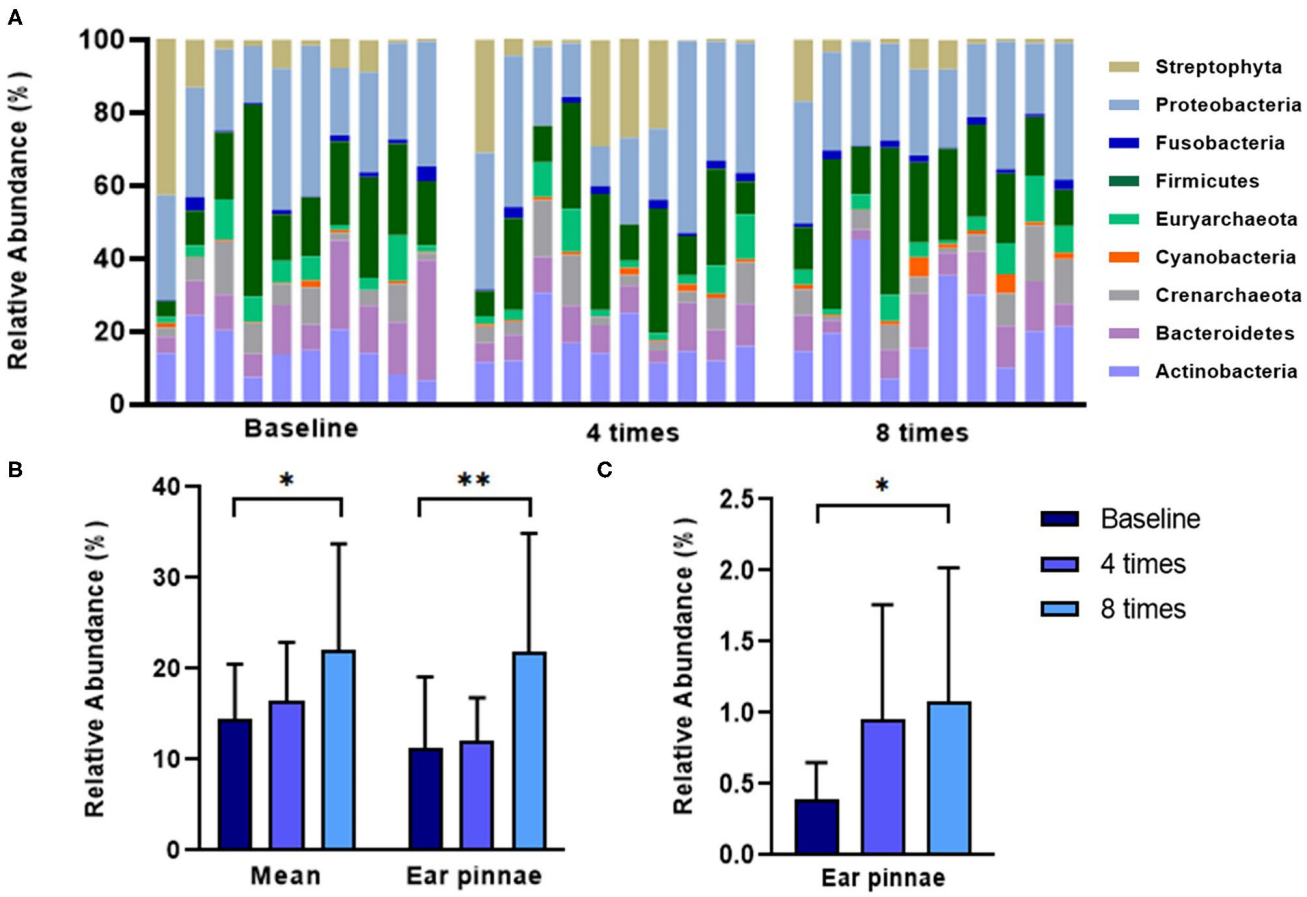
Change in the skin microbiome in course of excimer light therapy in allergic group at the phylum level. **(A)** Individual composition of bacterial phyla in the course of excimer light therapy in the allergic group. **(B)** Statistical analysis of the relative abundance of the phylum Actinobacteria. **(C)** Statistical analysis of the relative abundance of the phylum Cyanobacteria. Four times, fourth session of excimer light therapy in allergic group; eight times, end of the excimer light therapy in allergic group; baseline, before the start of excimer light therapy in the allergic group. All values are expressed as mean ± standard error. ^*^P < 0.05, ^**^P < 0.01.

Excimer light therapy increased the abundance of the phyla Actinobacteria and Cyanobacteria in the ear pinnae in the allergic group (Actinobacteria, 11.2 ± 7.9% to 21.8 ± 13.1%, *P* < 0.01; Cyanobacteria, 0.39 ± 0.3% to 1.0 ± 0.9%, *P* < 0.05; Figure 7). When analyzing the mean relative abundance of the phyla at all collected sites, excimer light therapy induced an increase in the phylum Actinobacteria in the allergic group (from 14.5 ± 6.0% to 22.0 ± 11.8%, *P* < 0.05; Figure 7).

### Analysis of α-Diversity of the Skin Microbiome

**Figure 8:**
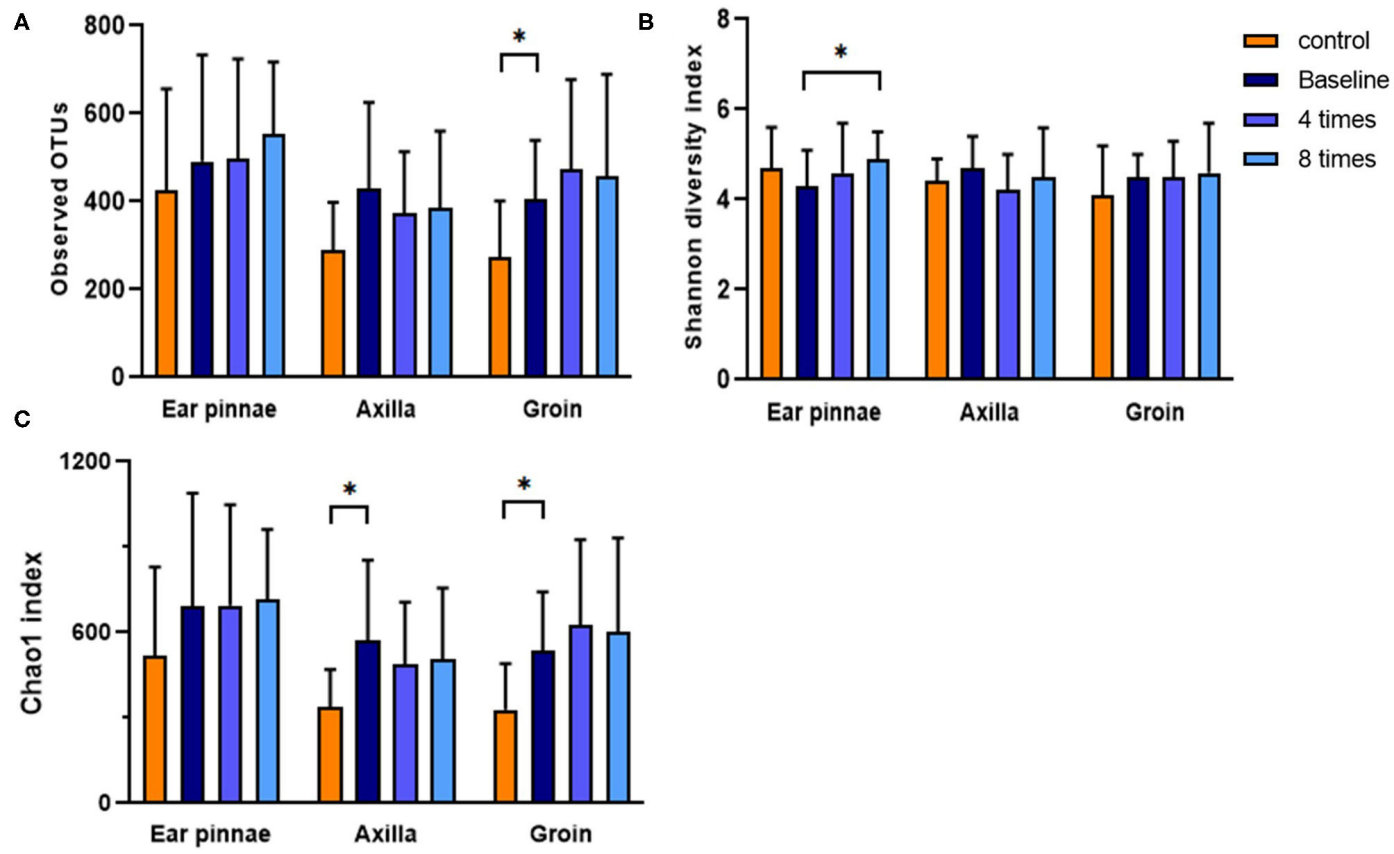
Analysis of diversity of the skin microbiome. Difference in the diversity of the skin microbiome between the control and allergic groups and change in the diversity of the skin microbiome after excimer light therapy in the allergic group. **(A)** observed OTUs. **(B)** Shannon diversity index. **(C)** Chao 1 index. Four times, fourth session of excimer light therapy in allergic group; eight times, end of the excimer light therapy in allergic group; baseline, before start of excimer light therapy in allergic group; OTUs, operational taxonomic units). All values are expressed as means ± standard error. ^*^P < 0.05.

Diversity was analyzed for 4,000 randomly selected sequences per sample. The observed Operational Taxonomic Units (OTUs, the observed bacterial species), Chao 1 index (estimated bacterial species richness), and Shannon diversity index (estimated bacterial diversity) were analyzed in the control and allergic groups. In the groin, the observed OTUs and Chao 1 index were significantly higher in the allergic group than in the control group (observed OTUs, allergic group, 406.2 ± 132.8, control group, 274.3 ± 126.8, *P* < 0.05; Chao 1 index, allergic group, 537.5 ± 206.4, control group, 328.3 ± 162.7, *P* < 0.05; Figure 8). In the axillary region, the Chao 1 index was significantly higher in the allergic group than in the control group (allergic group, 570.8 ± 282.2, control group, 336.3 ± 134.1, *P* < 0.05; Figure 8). Excimer light therapy induced a significant increase for the Shannon diversity index in the ear pinnae of the allergic group (from 4.3 ± 0.8 to 4.9 ± 0.6, *P* < 0.05; Figure 8).

### Analysis of *Staphylococcus* Species

**Figure 9:**
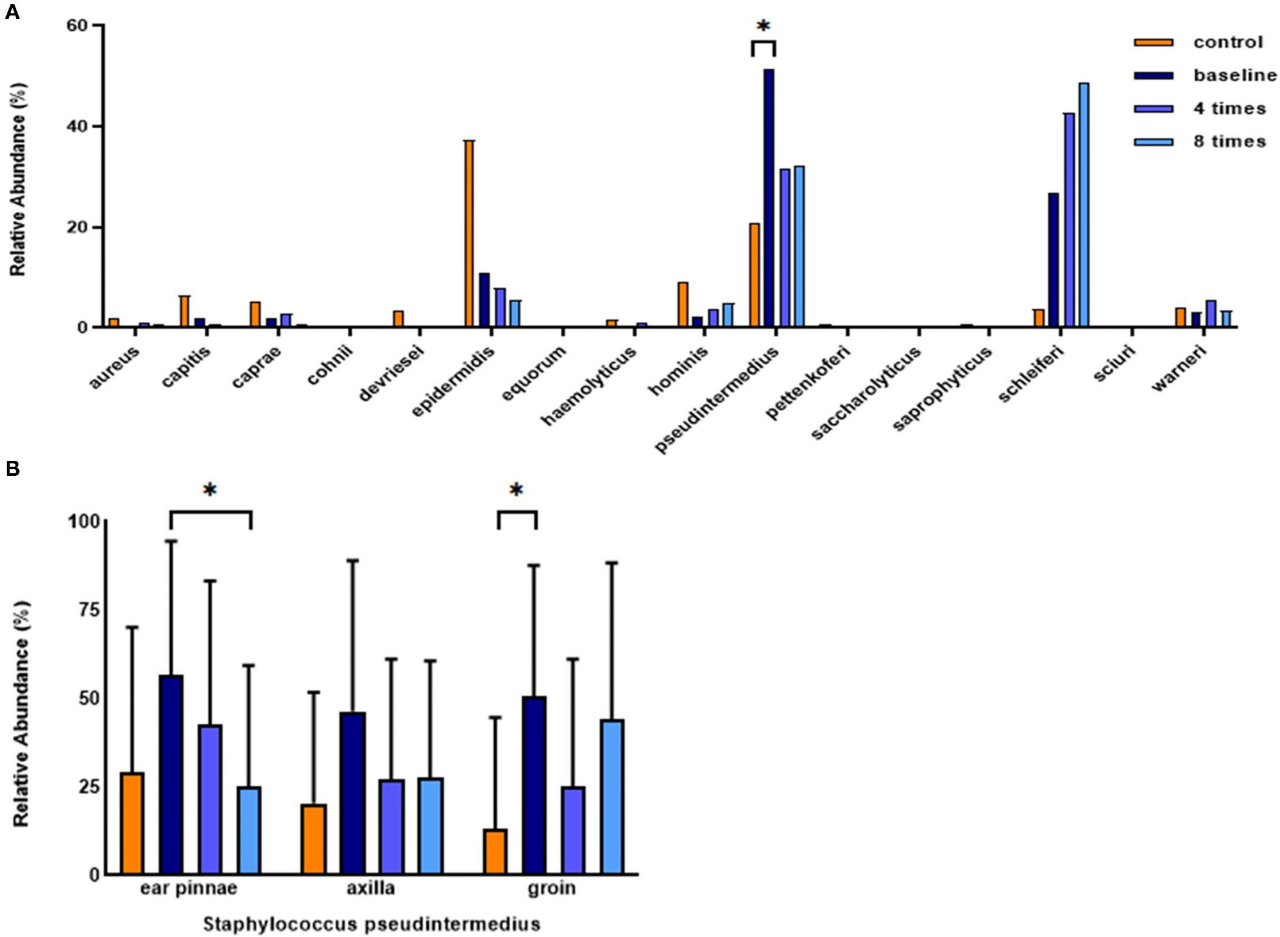
Analysis of *Staphylococcus* species and change in *Staphylococcus pseudintermedius* in the course of excimer light therapy. **(A)** Individual composition of *Staphylococcus* species in the control and allergic groups. **(B)** Statistical analysis of the relative abundance of *S. pseudintermedius*. Four times, fourth session of excimer light therapy in the allergic group; eight times, end of the excimer light therapy in the allergic group; baseline, before the start of excimer light therapy in allergic group. All values are expressed as mean ± standard error. ^*^P < 0.05.

In all analyzed sites, *Staphylococcus epidermidis* was the most abundant Staphylococcus species, followed by *S. pseudintermedius, S. hominis*, and *S. caprae* in the control group. In the allergic group, *S. pseudintermedius* was the most abundant Staphylococcus species, followed by *S. schleiferi, S. epidermis*, and *S. warneri* (Figure 9A). The mean relative abundance of *S. pseudintermedius* was higher in the allergic group than in the control group (allergic group, 51.3 ± 32.6%, control group, 20.9 ± 31.2%, P < 0.05; Figure 9A). Excimer light therapy resulted in an overall decrease in *S. pseudintermedius*. In the ear pinnae, the relative abundance of *S. pseudintermedius* decreased significantly in the allergic group after excimer light therapy (56.8 ± 37.7% to 25.2 ± 34.2%, *P* < 0.05; Figure 9B).

**Figure 10:**
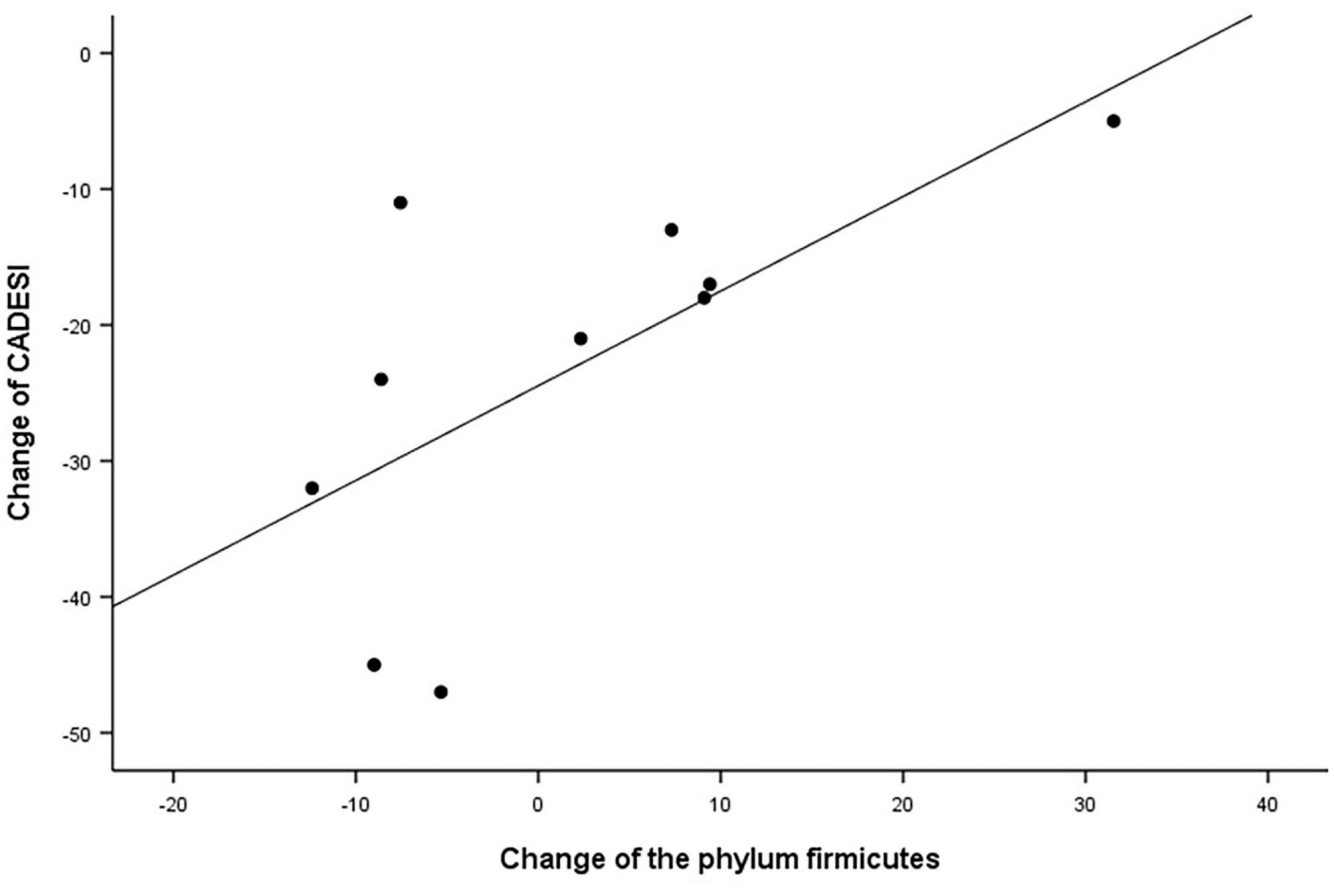
Correlation analysis. Correlation between the CADESI and change in relative abundance of the phylum Firmicutes in the allergic group after excimer light therapy. CADESI, canine atopic dermatitis extent and severity index.

In the correlation analysis between the clinical outcomes of therapy and the change in the skin microbiome and skin barrier function in the allergic group, the CADESI-4 had a significantly positive correlation with the phylum Firmicutes (*R* = 0.659, *P* < 0.05; Figure 10). Details regarding these correlations are provided in Supplementary Table 3.

## Discussion

In the present study, we examined the effect of topical 308-nm excimer light therapy for cAD on the skin microbiome, skin barrier function, and clinical outcomes. Excimer light therapy significantly altered the skin microbiome in cAD, thereby significantly increasing the relative abundance of the phyla Actinobacteria and Cyanobacteria. It significantly reduced clinical signs of cAD without inducing serious adverse effects (mild erythema). TEWL, a measure of skin barrier function, significantly decreased after phototherapy. Additionally, the treatment increased microbial diversity and decreased the relative abundance of *S. pseudintermedius*, which is associated with the severity of canine atopic dermatitis.

cAD is remarkably similar to human AD in terms of clinical, immunological, and pathophysiological factors (19, 20). AD is influenced by multiple factors such as genetics, immune responses, and the environment (10, 21). These factors also include an increase in IgE directed against environmental allergens (22), a predominance of T-helper 2 cells associated with regulatory T-cell deficiency (22, 23), an increase in the pruritic cytokine IL-31 (24), alteration of filaggrin metabolism, resulting in epidermal dysfunction (25), and bacterial colonization, particularly of *S. pseudintermedius*, in cAD (9). Bactericidal action, an anti-pruritic effect, the induction of T-cell apoptosis, and decrease in serum IgE levels have all been ascribed to excimer light treatment (26–29). The present study built on the findings of these preceding studies and showed that excimer light treatment significantly improved the clinical signs of cAD.

TEWL and SCH are parameters used to evaluate skin barrier function (30). TEWL increases in both lesional and non-lesional skin in cAD and continues to increase with the severity of cAD (10, 30). SCH of the lesional skin in cAD decreased compared with that of non-allergic skin in a healthy dog; however, a significant difference was not observed between non-allergic skin in a healthy dog and non-lesional skin in cAD (30). In this study, excimer light therapy was applied to dogs with cAD regardless of whether the AD lesion was present or not, and the phototherapy significantly improved TEWL, but not SCH. As aforementioned, though the phototherapy was performed in both lesional and non-lesional skin in cAD, significant improvement was confirmed only in TEWL.

In the skin microbiome analysis, an increase in the relative abundance of the phylum Cyanobacteria, which mainly populates moist environments, was indicative of a moisture content increase in the SC, corresponding to the results of AD in humans (7, 31). The abundance of the phylum Actinobacteria decreased among skin lesions in human AD, and that of Porphyromonas species, which belongs to this phylum, decreased in cAD lesions (10, 32). In this study, excimer light therapy increased the relative abundance of the phylum Actinobacteria in cAD, implying that the treatment induces normalization of the microbial dysbiosis in cAD.

Earlier reports differ in the descriptions of the microbial species diversity in cAD among healthy dogs. In a study analyzing the skin microbiome after experimentally inducing cAD in beagle dogs, there was no difference in the microbial diversity between those with cAD and those without cAD (9). In another study that analyzed the skin microbiome in client-owned dogs with cAD, microbial diversity was found to be lower in allergic dogs (10). Thus, it is somewhat surprising that this study showed a trend for increased diversity among dogs with cAD compared to healthy dogs. However, by comparison, the microbial α-diversity index lacks correlation with an AD diagnosis in humans. In human skin microbiome studies, the skin microbiome is affected by intrinsic factors such as age, ethnicity, and disease state, and extrinsic factors such as UV exposure, soil contact, and cosmetics (33–35). Therefore, further studies that consider the above factors are required. In the allergic group in our study, excimer light therapy increased the diversity of skin bacteria in the ear pinnae. This finding was consistent with the results of several previous studies related to AD treatment (10, 36). The increase in skin bacterial diversity in the ear pinnae shows that the effect of excimer light therapy in the pinnae was better than the other sites, which corresponds to the decrease in the relative abundance of S. *pseudintermedius* in the ear pinnae.

Staphylococcal antigens contribute to the deterioration and sustenance of AD (23). Staphylococcus species are related to AD severity in both human AD and cAD (37, 38). Relative abundance of S. *pseudintermedius* was richer in the allergic group than the control group. In the control group, S. *epidermis* was the most abundant *Staphylococcus* species. In a recent study, S. *epidermidis* was the second most abundant species in the control group (10). These results are thought to be caused by the small number of samples. Therefore, a large-scale study is needed. The relative abundance of *S. pseudintermedius* decreased after excimer light therapy, implying that excimer light therapy alleviates flare states of cAD, in agreement with findings of the previous studies in both human AD and cAD (7, 10). Contrary to the expectations, this study did not show a correlation between changes in the relative abundance of *S. pseudintermedius* and cAD severity or skin barrier function. Our results instead presented that the abundance of the phylum Firmicutes, to which the genus Staphylococcus belongs, correlated positively with the CADESI-4 (32). Further large-scale studies comparing the excimer light non-treated lesion in cAD to confirm the correlation with changes in the relative abundance of *S. pseudintermeidus* and cAD severity and skin barrier function are required. If a study of this nature cannot be conducted, another kind of bacteria need to be found that are a part of the Firmicutes phylum.

Excimer light therapy for cAD does not require any specialized skills and is a treatment that any veterinarian can perform easily. It takes a very short time, requiring a maximum of 15 min, even in large breeds. In addition, out of all the side effects of phototherapy reported in human AD, only mild erythema without pruritus was identified as a side effect in phototherapy with cAD, and even this was observed only during the first session of the excimer light therapy and spontaneously improved within 1 week without specific treatment. Mild erythema caused by phototherapy with cAD can be distinguished from the flaring of cAD since the erythema was the size of the excimer light beam window without pruritus. The combination of other allergy treatments was more effective than excimer light monotherapy; however, excimer light therapy also showed a therapeutic effect close to that of the combination with other allergy treatments. We recommend excimer light therapy for dogs with mild and moderate cAD. In dogs with severe cAD, an immunosuppressive agent or glucocorticoid administration is recommended rather than excimer light monotherapy, or combination therapy may be considered.

In addition to our small sample size, another limitation of this study is that the skin microbiome and skin barrier function analyses were performed without dividing the excimer light treated lesion and non-treated lesion in cAD. Therefore, further studies are needed with large-scale randomized patients, blinded operators, comparing the non-treated lesion in cAD. In conclusion, this study showed that excimer light therapy is a suitable, safe, and new therapeutic option for treating cAD. Furthermore, the therapy may be considered as a monotherapy or in combination with drug treatment for cAD, which also acts as a spontaneous animal model of AD.

## Data Availability Statement

The datasets presented in this study can be found in online repositories. The names of the repository/repositories and accession number(s) can be found in the article/Supplementary Material.

## Ethics Statement

This study was approved by the KonKuk University Institutional Animal Care and Use Committee (approval number: KU20057). Written informed consent was obtained from the owners for the participation of their animals in this study.

## Author Contributions

J-YP: conceptualization, formal analysis, investigation, resource, writing-original draft preparation, writing-review and editing, and visualization. S-MK: formal analysis, data curation, writing-review, and editing. J-HK: conceptualization, writing-original draft preparation, writing-review and editing, supervision, project administration, and funding acquisition. All authors contributed to the article and approved the submitted version.

## Funding

This study received funding from the Lameditech Corporation. The funder provided the necessary funds for the excimer instrument and experimental analysis (Grant No. S202009S00044). The funder was not involved in the planning of the study design, collection, analysis, interpretation of data, the writing of this article, or the decision to submit it for publication.

## Conflict of Interest

S-MK is employed by the KR LAB Bio incorporation. The remaining authors declare that the research was conducted in the absence of any commercial or financial relationships that could be construed as a potential conflict of interest.

## Publisher's Note

All claims expressed in this article are solely those of the authors and do not necessarily represent those of their affiliated organizations, or those of the publisher, the editors and the reviewers. Any product that may be evaluated in this article, or claim that may be made by its manufacturer, is not guaranteed or endorsed by the publisher.
