# Supplementary material for: Efficacy of Phototherapy With 308-nm Excimer Light for Skin Microbiome Dysbiosis and Skin Barrier Dysfunction in Canine Atopic Dermatitis
Source: Front Vet Sci. 2021 Dec 3;8:762961. doi: 10.3389/fvets.2021.762961 (PMC8677939; doi:10.3389/fvets.2021.762961)
Supplement: Supplementary file 1 [file Table_1.DOCX]

Supplementary Material

# Supplementary Figures and Tables

## Supplementary Tables ****1: Primer sequences for PCR****

| **V1-3 region** | **Sequence (5-------->3)** |
| --- | --- |
| 16SV13_Mi_F | TCGTCGGCAGCGTCAGATGTGTATAAGAGACAGAGAGTTTGATCCTGGCTCAG |
| 16SV13_N1_Mi_F | TCGTCGGCAGCGTCAGATGTGTATAAGAGACAGNAGAGTTTGATCCTGGCTCAG |
| 16SV13_N2_Mi_F | TCGTCGGCAGCGTCAGATGTGTATAAGAGACAGNNAGAGTTTGATCCTGGCTCAG |
| 16SV13_N3_Mi_F | TCGTCGGCAGCGTCAGATGTGTATAAGAGACAGNNNAGAGTTTGATCCTGGCTCAG |
| 16SV13_Mi_R | GTCTCGTGGGCTCGGAGATGTGTATAAGAGACAGATTACCGCGGCTGCTGG |
| 16SV13_N1_Mi_R | GTCTCGTGGGCTCGGAGATGTGTATAAGAGACAGNATTACCGCGGCTGCTGG |
| 16SV13_N2_Mi_R | GTCTCGTGGGCTCGGAGATGTGTATAAGAGACAGNNATTACCGCGGCTGCTGG |
| 16SV13_N3_Mi_R | GTCTCGTGGGCTCGGAGATGTGTATAAGAGACAGNNNATTACCGCGGCTGCTGG |

## Supplementary Tables 2: Individual skin barrier function/Clinical efficacy results

| ID-excimer times | Group | CADESI | PVAS | TEWL (ear) | TEWL (axilla) | TEWL (groin) | SCH (ear) | SCH (axilla) | SCH (groin) |
| --- | --- | --- | --- | --- | --- | --- | --- | --- | --- |
| 1 | Control | NA | NA | 7.2 | 7.5 | 9.1 | 21.2 | 13.4 | 10.8 |
| 2 | Control | NA | NA | 5.7 | 16.1 | 4.5 | 4.8 | 9 | 3 |
| 3 | Control | NA | NA | 13.1 | 9.6 | 9.1 | 4.2 | 8.8 | 4 |
| 4 | Control | NA | NA | 8.2 | 16.2 | 6.6 | 10.2 | 14.5 | 30.7 |
| 5 | Control | NA | NA | 10.4 | 6.1 | 4.9 | 11.8 | 16.8 | 11.2 |
| 6 | Control | NA | NA | 6.4 | 8.5 | 8.3 | 12.8 | 5.8 | 7.5 |
| 7 | Control | NA | NA | 6.8 | 8.5 | 6.2 | 16 | 10 | 4 |
| 8 | Control | NA | NA | 6.6 | 5 | 5.7 | 6.4 | 5 | 1.6 |
| 9 | Control | NA | NA | 10.6 | 6.6 | 8.4 | 6 | 6.3 | 9.7 |
| 10 | Control | NA | NA | 8.6 | 6.9 | 5.5 | 27.2 | 27.4 | 27.2 |
| 11-baseline | Allergy | 85 | 7 | 29.4 | 109.8 | 53.8 | 16 | 11.3 | 9 |
| 12-baseline | Allergy | 28 | 8 | 13.9 | 59.7 | 14.8 | 5.5 | 11 | 2.7 |
| 13-baseline | Allergy | 110 | 6 | 30.8 | 21.7 | 32.2 | 18 | 4 | 4 |
| 14-baseline | Allergy | 102 | 5 | 26.4 | 113.7 | 97.5 | 5 | 4 | 8.5 |
| 15-baseline | Allergy | 45 | 9 | 106.4 | 14.4 | 11.7 | 5.2 | 8.2 | 5.2 |
| 16-baseline | Allergy | 34 | 7 | 6.4 | 98.3 | 12.7 | 13.4 | 13.4 | 12.2 |
| 17-baseline | Allergy | 40 | 8 | 21 | 17.8 | 15.2 | 4 | 6.3 | 4.8 |
| 18-baseline | Allergy | 49 | 8 | 19 | 22.5 | 17.4 | 3.2 | 3 | 4 |
| 19-baseline | Allergy | 129 | 5 | 61.2 | 108.7 | 24 | 24.2 | 43.4 | 9.8 |
| 20-baseline | Allergy | 21 | 7 | 16.7 | 16.2 | 11.1 | 4.8 | 7.3 | 3.3 |
| 11-4 times | Allergy | ND | ND | 18.2 | 41.4 | 6.2 | 11.3 | 17.8 | 17.5 |
| 12-4 times | Allergy | ND | ND | 11.3 | 82 | 10.9 | 8 | 15 | 8.3 |
| 13-4 times | Allergy | ND | ND | 16.6 | 24.5 | 14.8 | 21.2 | 17.6 | 22.6 |
| 14-4 times | Allergy | ND | ND | 26.7 | 97.4 | 6.5 | 10.6 | 10.5 | 9.4 |
| 15-4 times | Allergy | ND | ND | 12 | 16.2 | 9.4 | 4 | 6 | 4.4 |
| 16-4 times | Allergy | ND | ND | 9.8 | 22.3 | 16.7 | 5.3 | 7.6 | 8.4 |
| 17-4 times | Allergy | ND | ND | 17..9 | 15.9 | 13.9 | 2 | 3.3 | 4.4 |
| 18-4 times | Allergy | ND | ND | 15.8 | 14.7 | 14.4 | 5 | 5.5 | 5.3 |
| 19-4 times | Allergy | ND | ND | 46.7 | 63.9 | 22.3 | 38.6 | 18 | 10.2 |
| 20-4 times | Allergy | ND | ND | 12.3 | 10.5 | 8.1 | 7 | 6.6 | 8 |
| 11-8 times | Allergy | 72 | 4 | 17.9 | 14.1 | 29.6 | 16.8 | 17.5 | 15.2 |
| 12-8 times | Allergy | 23 | 2 | 8.1 | 14.1 | 13.5 | 9 | 3.3 | 5 |
| 13-8 times | Allergy | 63 | 4 | 15.8 | 22.4 | 14.9 | 28.2 | 17.8 | 12.2 |
| 14-8 times | Allergy | 70 | 1 | 8.6 | 85.3 | 8.6 | 4.8 | 5 | 7 |
| 15-8 times | Allergy | 28 | 3 | 16.4 | 7.9 | 6.5 | 2 | 3.3 | 3.5 |
| 16-8 times | Allergy | 16 | 4 | 4.5 | 12 | 11.3 | 11.3 | 13.3 | 8.6 |
| 17-8 times | Allergy | 19 | 6 | 13.7 | 12.3 | 12.9 | 8.4 | 6 | 17.3 |
| 18-8 times | Allergy | 25 | 5 | 12.8 | 12.9 | 7.1 | 15 | 4 | 3.8 |
| 19-8 times | Allergy | 84 | 4 | 25 | 46 | 12.1 | 21.8 | 11.8 | 34.4 |
| 20-8 times | Allergy | 10 | 3 | 5 | 7.7 | 5.4 | 3 | 2 | 2 |
| 4 times, at the 4 times of excimer light therapy in allergic group; 8 times, end of the excimer light therapy in allergic group; baseline, before start of excimer light therapy in allergic group; NA, not available; ND, not done; SCH, stratum corneum hydration; TEWL, trans-epidermal water loss. | | | | | | | | | |

## Supplementary Table 3: Correlation of clinical outcomes, skin barrier function and skin microbiome

|  | CADESI-4 | PVAS | TEWL | SCH |
| --- | --- | --- | --- | --- |
| TEWL | r=0.095  P=0.795 | r=0.098  P=0.787 |  | r=0.373  P=0.289 |
| SCH | r=-0.366  P=0.298 | r=0.421  P=0.225 | r=0.373  P=0.289 |  |
| Actinobacteria | r=-0.478  P=0.162 | r=0.493  P=0.147 | r=0.595  P=0.070 | r=0.336  P=0.343 |
| Firmicutes | r=0.662  P=0.037 | r=-0.592  P=0.071 | r=0.016  P=0.965 | r=-0.185  P=0.609 |
| Cyanobacteria | r=0.149  P=0.681 | r=-0.405  P=0.246 | r=0.127  P=0.127 | r=-0.202  P=0.577 |
| *Staphylococcus pseudintermedius* | r=0.560  P=0.092 | r=-0.308  P=0.387 | r=-0.401  P=0.250 | r=-0.459  P=0.183 |
| Observed OTUs | r=0.307  P=0.389 | r=-0.079  P=0.827 | r=-0.229  P=0.525 | r=-0.598  P=0.068 |
| Chao 1 index | r=0.323  P=0.363 | r=-0.075  P=0.837 | r=-0.238  P=0.508 | r=-0.566  P=0.088 |
| Shannon index | r=0.161  P=0.657 | r=0.234  P=0.515 | r=-0.156  P=0.667 | r=-0.269  P=0.453 |
| CADESI-4, canine atopic dermatitis extent and severity index version 4; PVAS, pruritus visual analog scale; SCH, stratum corneum hydration; TEWL, transepidermal water loss | | | | |
